# High-Resolution Imaging and Interpretation of Three-Dimensional RPE Sheet Structure

**DOI:** 10.3390/biom15081084

**Published:** 2025-07-26

**Authors:** Kevin J. Donaldson, Micah A. Chrenek, Jeffrey H. Boatright, John M. Nickerson

**Affiliations:** 1 Department of Ophthalmology, Emory University, Atlanta, GA 30322, USA; kevin.donaldson@emory.edu (K.J.D.); micah.chrenek@emory.edu (M.A.C.); jboatri@emory.edu (J.H.B.); 2 Atlanta Veterans Administration Center for Visual and Neurocognitive Rehabilitation, Decatur, GA 30033, USA

**Keywords:** retinal pigment epithelium, RPE, 3D reconstruction, cell segmentation, EMT, multinucleation, blood–retina barrier

## Abstract

The retinal pigment epithelium (RPE), a monolayer of pigmented cells, is critical for visual function through its interaction with the neural retina. In healthy eyes, RPE cells exhibit a uniform hexagonal arrangement, but under stress or disease, such as age-related macular degeneration (AMD), dysmorphic traits like cell enlargement and apparent multinucleation emerge. Multinucleation has been hypothesized to result from cellular fusion, a compensatory mechanism to maintain cell-to-cell contact and barrier function, as well as conserve resources in unhealthy tissue. However, traditional two-dimensional (2D) imaging using apical border markers alone may misrepresent multinucleation due to the lack of lateral markers. We present high-resolution confocal images enabling three-dimensional (3D) visualization of apical (ZO-1) and lateral (α-catenin) markers alongside nuclei. In two RPE damage models, we find that seemingly multinucleated cells are often single cells with displaced neighboring nuclei and lateral membranes. This emphasizes the need for 3D analyses to avoid misidentifying multinucleation and underlying fusion mechanisms. Lastly, images from the NaIO_3_ oxidative damage model reveal variability in RPE damage, with elongated, dysmorphic cells showing increased ZsGreen reporter protein expression driven by EMT-linked CAG promoter activity, while more regular RPE cells displayed somewhat reduced green signal more typical of epithelial phenotypes.

## 1. Introduction

The retinal pigment epithelium (RPE) is a monolayer sheet of cells in the eye that forms a barrier between the neural retina and the choroid. The RPE plays a critical role in retinal homeostasis through a myriad of functions, primarily physically and metabolically supporting the adjacent rod and cone photoreceptor cells of the neural retina [1,2,3,4,5].

In healthy RPE tissue, the apical surfaces of individual cells in the sheet present with the appearance of regular hexagons when viewed en face. Each RPE cell typically contacts six immediately adjacent neighbor RPE cells, establishing the outer blood–retina barrier. Mature RPE cells are terminally differentiated, generally quiescent, and contact-inhibited in adult humans and rodent models. Fewer than 0.1% are positive for cell division markers like Ki67 and BrdU, and these dividing cells are typically found in the periphery rather than the center [6]. Under severe stress, RPE cells may die, proliferate, or re-differentiate, as seen in cell culture [7,8], after laser burns or in conditions like PVR and AMD [9]. In unusual or diseased states, RPE cells can undergo senescence [10], epithelial–mesenchymal transition (EMT) [11], or simple cell division [12]. During normal development, RPE cell numbers increase about fourfold from E15 to P15 in healthy C57Bl/6J mice [13,14,15]. After this period, RPE cell numbers remain relatively stable during normal aging [15] until extreme old age (>2 years old) [16].

Studies examining RPE cell structure have revealed correlations between abnormal morphology and multiple aging and blindness disorders, such as age-related macular degeneration (AMD) and other inherited retinopathies [17,18,19,20,21]. Under stressful conditions, the RPE sheet, which is generally highly robust, can undergo numerous signs of damage, exhibiting both functional and morphological changes. While the RPE sheet can lose barrier functions, it appears to preserve the monolayer barrier by employing multiple reconfiguration processes. For instance, dead or dying RPE cells can be extruded in a classical purse-string mechanism, and surrounding cells appear to fill in by changing shapes in a pie-like pattern [22,23].

Under aging conditions, as well as after severe stress, some RPE cells become much larger, but it is unclear how this happens. Is it a single cell that gains size, or possibly two cells fused together, resulting in a larger single cell? Additionally, it is unclear how these stressed or damaged cells are able to move. Is there a modification to Bruch’s membrane itself, such that cells translocate by traversing laterally on the surface of a stationary Bruch’s membrane, or is it the reverse, where perhaps Bruch’s membrane is modified itself and the attached anchored cells move as the membrane does?

An additional observation of these cells under abnormal conditions is that some appear to be multinucleate. While it is well established that rodent RPE are uni- or binucleate, it is less clear if, or how, a single RPE cell might acquire additional nuclei beyond two. The appearance of three or more nuclei within a single RPE cell may result from the displacement of nuclei from adjacent cells moving above or below the focal plane. This phenomenon, coupled with parallax effects inherent in standard en face imaging techniques, can create the illusion of multinucleation within individual RPE cells. Alternatively, pathological circumstances might lead to cell fusion, which may offer cells the opportunity to share resources and thus save the remaining damaged cells. Cell fusion may also be a potential mechanism that could enable thinner cells to cover more of Bruch’s membrane via an increase in surface area without requiring an increase in cell number. There is good evidence to support this model in aging and potential stress models [24], thus it becomes important to ensure that we accurately identify which nuclei belong to which cell and whether or not multinucleate cells are true hallmarks of abnormal RPE sheet or potential imaging artifact.

A potential confound of visual RPE nuclei localization in standard en face images is that the choroid has many nucleated cells. Show-through of endothelial cells can occur if the density of pigment granules of the RPE or melanocytes is low, which might occur in disease and therefore it is essential that we do not mistakenly count any of these nuclei when determining RPE nuclei counts per cell. Thus, it is critical to accurately observe the location and shape of nuclei in order to establish whether a given nucleus is really a nucleus of the RPE or another confounding layer in a flatmount. Standard imaging of ex vivo RPE sheet preparations typically involves simultaneous immunofluorescent staining of apical cell border proteins, such as ZO-1, and nuclei with different wavelength fluorophores. Images acquired with a confocal microscope are usually at a lower magnification (10–20× objective) as global patterning is of initial interest in multifield stitched images. In some experiments, multiple images in the z-axis are acquired (z-stacks), which are then converted to 2D maximum intensity projections (MIPs) by collapsing the z-axis. In flatmount preparations, the z-axis closely follows the apical to basal axis (A-B axis) of the RPE cell but with some imperfections. These imperfections may arise from technical or preparation artifacts, such as slight hills and valleys in the otherwise flat RPE sheet. However, certain RPE cells may also exhibit inherently irregular 3D shapes, including non-orthogonal orientation of the apical–basal axis. Generating 2D MIPs from z-stacks of tissue mounted at standard en face flatmount angles results in loss of 3D information, which can lead to misinterpretation of subcellular localization (e.g., nuclei). This loss may introduce parallax artifacts, where nuclei from neighboring cells appear within the borders of a central cell if those neighboring cells have expanded apical surfaces without corresponding displacement along the z-axis. For experiments aiming to assign nuclei to their respective cells for downstream analyses, preserving 3D data enables more accurate localization. While some 3D ultrastructure imaging of RPE cells has been achieved via serial block-face scanning electron microscopy (SBF-SEM) [25], this method still presents a technical barrier for many labs as well as not being suited for simultaneous whole-eye image acquisition used in disease phenotyping.

A high magnification (60×) 3D rendering of the RPE sheet with fluorescent staining of the apical cell borders, basal, and lateral faces of individual RPE cells can help us decode patterns associated with normal RPE cell properties, as well as those in stressed or damaged conditions. Additionally, when imaged with modern, automated confocal microscopes, entire eye flatmount preparations can be obtained for large scale pattern recognition before subsequent imaging of specific dysmorphic cells of interest.

Thoughtful interpretation of 3D ultrastructure imaging should also be applied to other RPE cell dysmorphia. We and others find that RPE cells undergo progressive degeneration in patients [1,2,4,5] and in animal models of retinal damage or disease [26,27,28], exhibiting characteristics of dedifferentiation reminiscent of epithelial-to-mesenchymal transition (EMT; reviewed in [29]. Experiments with cultures of RPE cells or explants also show that when stressed, RPE cells appear to dedifferentiate via EMT-like processes [26,29,30,31,32,33,34].

The existence of RPE “epithelial-to-mesenchymal transition” (RPE EMT) may imply that RPE cells lose apical–basal polarity, normal intercellular adhesion, and outer blood–retina barrier functions. RPE cells might become mesenchymal cells because they have begun to move and migrate and invade the neural retina. This transition might include the loss of E- and P-cadherin and increase expression of putative “mesenchymal markers” including SMA, Vimentin, Snail, Twist, and Slug. Here we image complete RPE monolayers after NaIO_3_ tail-vein injections, illustrating stress responses of RPE cells in different locations demonstrating variable pleomorphic and polymorphic responses that differ radially. The degree of response is also indicated by apparent changes in activity of a CAG promoter driving ZsGreen expression consistent with increased activity reminiscent of changing from an epithelial level of low expression to a higher level of activity like that found in mesenchymal cells [35].

“RPE EMT” may represent a wound-healing response, potentially involving fibrosis, triggered by external signals such as growth factors, cytokines, and extracellular matrix components. While it remains uncertain whether “RPE EMT” constitutes a true epithelial–mesenchymal transition, it likely shares commonalities with other EMT processes, suggesting that therapies targeting EMT in other contexts could be effective for RPE-related diseases and wound healing.

In the present experiments, damage to RPE cells in mice was modeled in vivo using three established methods: light damage, subretinal injections causing retinal detachment, and sodium iodate treatment. These models were selected for their frequent use in the field, ability to induce varying degrees of damage, speed, and relevance to current clinical needs for treating RPE damage in humans. Although none of these models are ideal, each is sufficient for the experimental goals and interpretations discussed here. Photic injury [36] involves exposing heavily pigmented C57Bl/6J mice to lighting conditions that cause oxidative damage to photoreceptors and RPE cells [16,37]. This acute model mimics human conditions such as solar retinopathy or exposure to intense light sources. Subretinal injections that create temporary retinal detachments in the form of large blebs result in RPE damage [38,39,40] while low-dose sodium iodate selectively and rapidly damages RPE cells when administered via tail-vein injection [28,41].

Details on the implementation of these models are provided in the Section 2, with references to previous publications.

## 2. Methods

### 2.1. Animal Models of RPE Damage

All mouse handling procedures and care were approved by the Emory Institutional Animal Care and Use Committee (protocol PROTO201700013) and followed the ARVO Statement for the Use of Animals in Ophthalmic and Vision Research. Adult (postnatal day 90) male C57BL6/J mice were obtained from The Jackson Laboratory (Bar Harbor, ME, USA) and were housed under a 12:12-h light–dark cycle. Following our previously published protocol [42], one mouse was exposed to toxic levels of light which caused light-induced retinal degeneration (LIRD). Subretinal injection surgeries were performed in additional mice, causing a temporary retinal detachment and subsequent RPE sheet disruption [39]. Animals were sacrificed and tissue harvested a few days after the respective procedures. For sodium iodate-induced RPE damage, we employed an inducible VMD2-Cre driver line [43] (JAX strain# 032910) crossed to a CAG-Lox-Stop-Lox-ZsGreen mouse line [44] (JAX strain# 007906) all on the C57BL/6J background. Starting at P50, mice were fed rodent chow containing doxycycline (200 mg/kg Doxycycline) from Bio-Serv (Flemington, NJ, USA; cat# S3888) for 2 weeks. This cross yielded RPE cells that were indelibly tagged with ZsGreen expression. Any cells that were originally normal, mature RPE in phenotype and subsequently “transdifferentiated” into another cell type remain permanently marked by ZsGreen fluorescence, regardless of their phenotypic fate. Tail-vein injections were conducted as previously described [28].

### 2.2. Tissue Preparation and Immunofluorescent Labeling

**Figure 1:**
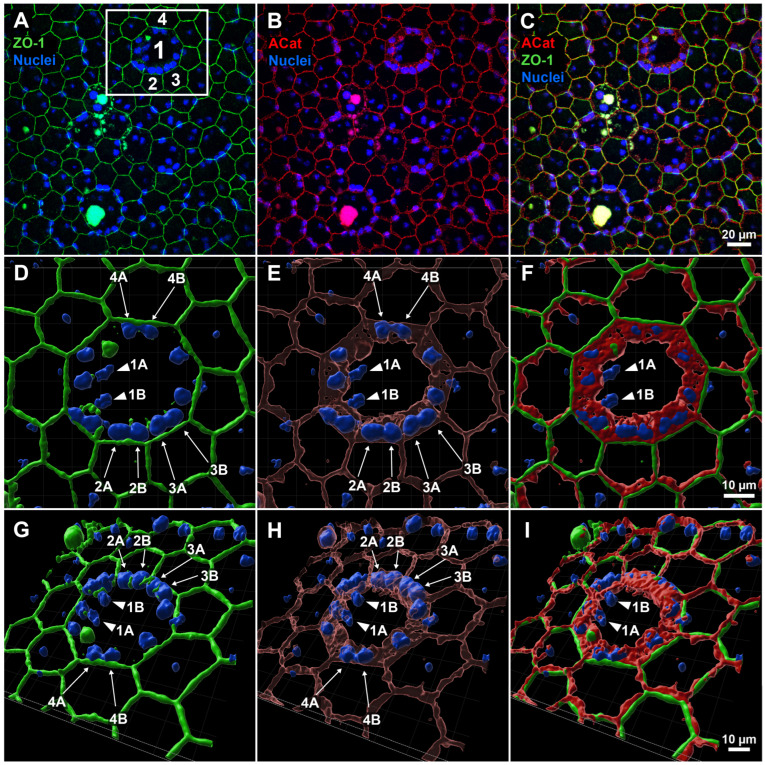
Three-dimensional reconstruction of apical and lateral structure of enlarged central and surrounding cells in RPE sheet following LIRD. (**A**–**C**) Original field of view (FOV) from whole-mount RPE confocal imaging, showing labeling for apical junction marker ZO-1 (green), lateral junction and perimeter marker α-catenin (red), and nuclei (blue). A region of interest (white box in (**A**)) highlights a central abnormal cell (1) with two nuclei (1A, 1B) and surrounding neighboring cells (2, 3, 4). (**D**–**F**) En face 3D reconstructions of the ROI reveal that nuclei of adjacent cells (2A, 2B; 3A, 3B; 4A, 4B) are clearly enclosed by α-catenin at the lateral borders of their respective cells, whereas the central nuclei (1A, 1B) appear more exposed and are not similarly embedded within α-catenin-defined junctions. (**G**–**I**) 3D views from a basal perspective further confirm the spatial arrangement of nuclei and α-catenin, supporting correct attribution of each nucleus to individual cells and excluding the presence of more than two nuclei per RPE cell. Video S1 (supplemental) provides additional viewing angles and magnification of this specific FOV.

**Figure 2:**
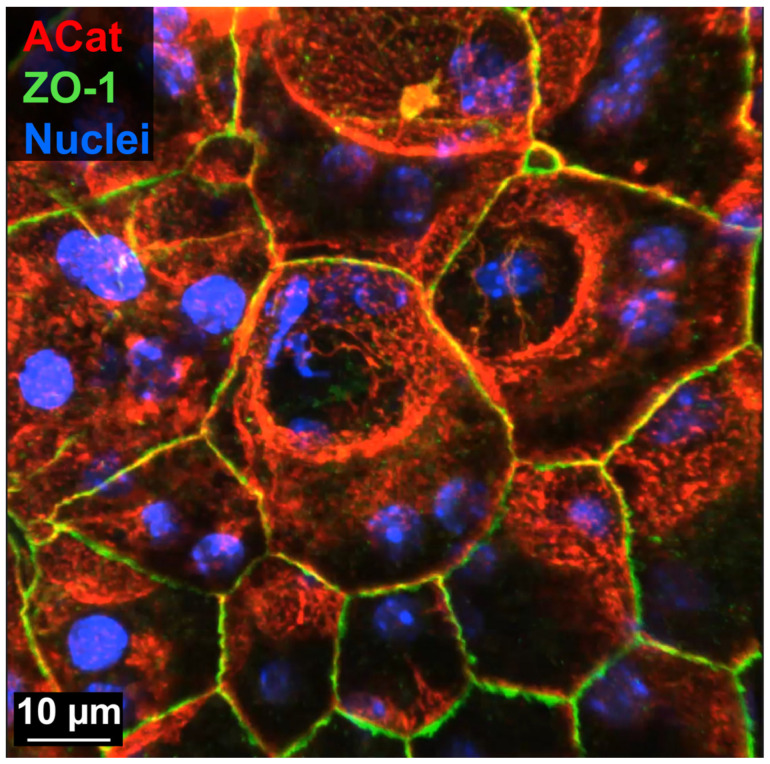
This static image is taken from Video S2 in the supplement. Multi-angle view of RPE cell dysmorphia following subretinal injection (SRI)-induced retinal detachment. Three-dimensional video of confocal z-stack (100× magnification) showing ZO-1 cell perimeter (green), α-catenin lateral and perimeter (red) and nuclear (blue) labeling. Vast changes in individual RPE cell size, shape, orientation and alignment are visible during recovery from SRI-induced retinal detachment. Again, large, seemingly multinucleate cells are shown to contain less nuclei than expected when using ZO-1 perimeter staining and typical apical viewing, instead of lateral labeling of cell membranes and alternative viewing angles in 3D.

Whole-eye RPE flatmounts (FMs) were prepared following our previously published protocols [45,46] with some minor modifications (Figure 1, Figure 2, Figure 4 and Figure 5; Video S1 and S2). Eyes were fixed in Z-Fix (Anatech Ltd., Battle Creek, MI, USA) for 10 min, and then washed three times with Hank’s Balanced Salt Solution (HBSS; Cat. #14025092, Gibco by Life Technologies, Grand Island, NY, USA). Eyes were stored at 4 °C for up to 24 h before being dissected. Following the removal of the iris and neural retina, four radial cuts were made to produce four RPE–scleral flaps with additional smaller relief cuts to aid with flattening of the tissue. FMs were then mounted RPE side up, on conventional microscope slides to which a silicon gasket had been applied (Grace Bio-Labs, Bend, OR, USA).

The FMs were rinsed with HBSS, then incubated in blocking buffer consisting of 1% BSA (Sigma, St. Louis, MO, USA) in 0.1% Triton X-100 (Sigma) HBSS solution for 1 h at room temperature. Primary incubation occurred overnight at room temperature (1:100 anti-ZO-1, Cat. #MABT11, MilliporeSigma, Burlington, MA, USA); 1:500 anti-CTNNA1 (α-catenin) [EP1793Y], Cat. #ab51032, Abcam, Cambridge, MA, USA). Following washes with 0.1% Triton-X-100 in HBSS, FMs were incubated with secondary antibodies (Alexa Fluor 488,1:1000 donkey anti-rat immunoglobulin G, Cat. #A21208, Thermo Fisher Scientific, Waltham, MA, USA; Alexa Fluor 568, 1:1000 goat anti-rabbit immunoglobulin G, Cat. #A11036, Thermo Fisher Scientific) overnight at room temperature. FMs were washed with Hoechst 33258 nuclear stain in 0.1% Triton X-100 in HBSS three times, followed by two washes with only wash buffer, and then mounted with Fluoromount-G (Cat. #17984-25, Electron Microscopy Sciences, Signal Hill, CA, USA), coverslipped, and allowed to set overnight.

**Figure 3:**
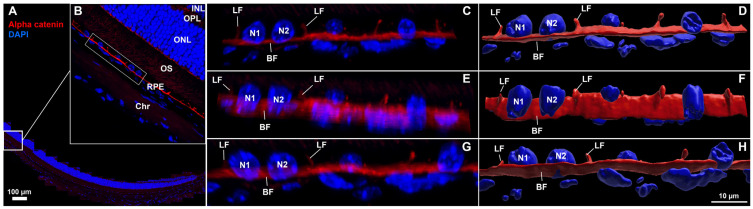
Confocal images and corresponding 3D reconstructions of the retina–RPE–choroid with lateral and basal surface labeling of RPE cell membranes in normal mouse eyes. (**A**,**B**) Region of interest (ROI) selection from a 5 µm thick cross-section labeled for α-catenin (red) and nuclei (blue), scale bar = 100 µm. A 3-cell-wide region (bounding box in (**B**)) was isolated to provide alternate-angle views (**C**,**E**,**G**) and corresponding 3D reconstructions (**D**,**F**,**H**). RPE nuclei (N1, N2) were morphologically distinct from choriocapillaris nuclei which were visible below the basal α-catenin labeling. Video S3 (in Supplemental Materials) of reconstructed ROI rotated for viewing from multiple angles corresponds to (**D**,**F**,**H**) (LF = lateral face, BF = basal face, INL = inner nuclear layer, OPL = outer plexiform layer, ONL = outer nuclear layer, OS = outer segments of photoreceptors, RPE = retinal pigment epithelium, Chr = choroid).

Ocular cross-sections (Figure 3, Video S3) were prepared using a modification of the freeze substitution technique described by Sun et al. [47]. Specifically, after enucleation, eyes were rapidly frozen in 10 mL of 3% acetic acid in methanol that had been prechilled in dry ice. The eyes were fixed and dehydrated at −80 °C for 4 days, cleared with methanol and xylenes, and embedded in paraffin. 5 µm sections were cut and immunostained with anti-α-catenin antibody (same antibody as FM preparation), counterstained with Hoechst 33342. 

### 2.3. Confocal Microscopy and 3D Image Visualization

Whole-eye FMs were imaged in their entirety at lower magnification using either a Nikon Ti microscope with C1 confocal scanner and Nikon Plan Apo 10× dry DIC N1 objective (NA = 0.45 WD = 4.0 mm) at 1024 × 1024 resolution or a Nikon Ti2 microscope with A1R confocal scanner and Nikon Plan Apo Lambda 20x dry lens (NA = 0.75 WD = 1.0 mm) at 2048 × 2048 resolution, (Nikon Instruments Inc., Melville, NY, USA). Sufficient total z-stack thickness to capture entire RPE cells throughout the FM was used (~75 µM). Regions of Interest (ROIs) containing dysmorphic cells were then reimaged at higher magnification (60× and 100× objective lenses, 1024 × 1024 resolution) with appropriate z-slice spacing to satisfy the Nyquist requirement for 3D capture and viewing. 100×: 9.63 µM range, 0.13 µm step size.

Ocular cross-sections were collected using a Nikon Ti2 microscope with A1R confocal imager and a 40× silicone oil objective with 2.5× zoom (Nikon Plan Apo Lambda S 40× silicone oil immersion lens, N = 1.25, WD = 0.30 mm with correction collar set at 0.17) and 0.225 µm Z-stack step size at 2048 × 2048 resolution. The area imaged was approximately 250–750 µm from the posterior pole.

Imaris (version 10.1, Bitplane, Oxford, UK) software was used to isolate and post-process ROIs from raw confocal files as well as generate videos highlighting cell membrane, internal α-catenin structure, and nuclear position from multiple viewing angles. While Imaris is a commercial product, ImageJ2 (version: 2.16.0/1.54p) and relevant plugins (such as 3D ImageJ Suite [48]) offers similar capabilities for handling 3D information.

### 2.4. Morphometric Analyses

A single field of view (FOV) was selected from a sodium iodate-treated RPE whole mount, capturing cells near an atrophic region. Cells were manually segmented and categorized as either EMT-like or normal based on morphology and location relative to the damage border. Only cells with clearly defined borders, as identified by ZO-1 labeling (red), were included. Within each group, cells were selected from similar radial distances from the atrophic zone to minimize positional bias.

Morphological parameters—including cell area, circularity, and aspect ratio—were measured in FIJI2 (ImageJ2 v1.52p) [49] using the freehand selection tool and ROI Manager. A total of 30 EMT-like and 30 normal RPE cells were analyzed. Mean values and standard errors were calculated, and statistical comparisons were performed using unpaired two-tailed *t*-tests. Graphs were generated in GraphPad Prism (version 10, GraphPad Software, San Diego, CA, USA), using box-and-whisker plots showing the median, interquartile range, and full data range (min to max).

## 3. Results

**Figure 4:**
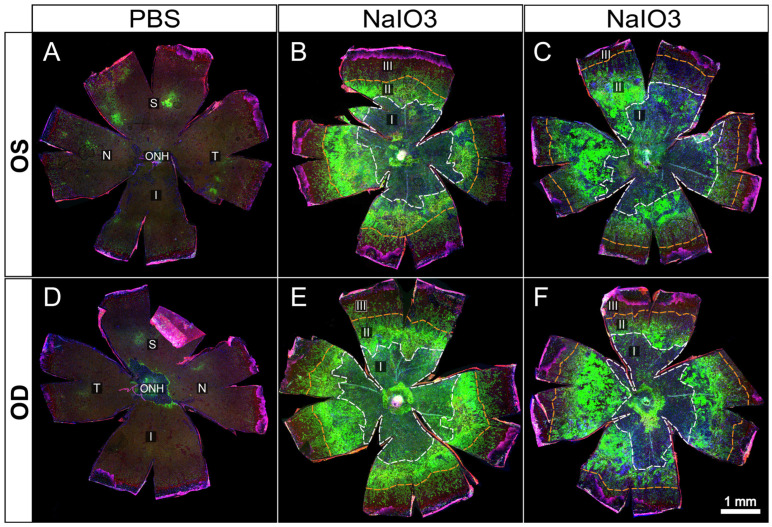
Representative examples of whole-eye flatmounts prepared 8 days following control (PBS) or NaIO_3_ tail-vein injections. Drastically altered RPE morphology was observed in both eyes of animals following intravenous injection of NaIO_3_ (**B**,**C**,**E**,**F**) but not in those injected with only PBS vehicle (**A**,**D**). Of note is a general gradient of cellular stress severity, originating from the optic nerve head. A large central atrophic lesion (I) is surrounded by a ring-like border region composed of cellular debris and aggregates (II) (bright green = ZsGreen expression driven by CAG promoter solely in RPE cells or descendants), most likely dying RPE cells. Continuing radially, an EMT-like response is seen characterized by elongation of RPE cells before transitioning to unaffected peripheral cells (III).

**Figure 5:**
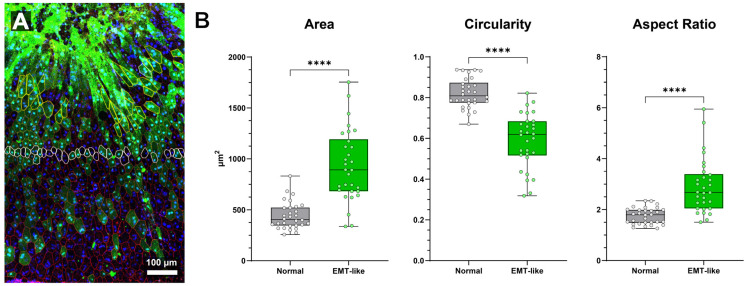
Comparison of normal and EMT-like class morphology in NaIO_3_-damaged RPE sheet (**A**) Region of interest of “RPE-EMT” transitional region following NaIO_3_ administration. Extremely high expression of ZsGreen linked to CAG promoter activity is evident in regions where cell morphology is severely abnormal (bright green top 1/3 of image) and apical cell border delineation is non-existent (red, ZO-1). Adjacent, intact cells are elongated before transitioning to more regular morphology further from the atrophic area. (**B**) Quantitative comparison of cell area, circularity, and aspect ratio between EMT-like and normal RPE cells (*n* = 30 per group). EMT-like cells are significantly more elongated, with increased aspect ratio and cell area, and reduced circularity (**** = *p* < 0.0001 for all comparisons, unpaired *t*-test). Box-and-whisker plots show the median, interquartile range, and full data range (min to max). Data are plotted for individual cells.

We present images, reconstructions, and videos generated from high magnification confocal z-stacks of two damage-model RPE sheets highlighting 3D ultrastructure of individual cells. Specifically, we selected ROIs that exhibit a heterogeneous mix of normal and dysmorphic cells and include larger than normal, multinucleate cells (Figure 1, Videos S1 and S2). Extending the visualization of conventionally sectioned ocular tissue, we also present 3D images, reconstructions and videos of lateral and basal RPE cell surfaces (Figure 2, Video S3). Finally, we show both local and global disruptions in the RPE sheet reminiscent of EMT in entire mouse eyes following induced RPE loss via intravenous tail-vein NaIO_3_ injections (Figure 4 and Figure 5).

To illustrate our argument that nuclear misattribution may occur in dysmorphic RPE cells following damage, we selected a single field of view (FOV) from an LIRD-damaged whole-eye RPE sheet stained for the apical marker ZO-1, apical and lateral marker α-catenin, and nuclei (Figure 1). Specifically, we looked for a region of interest (ROI) which contained both enlarged, seemingly multinucleate cells in close approximation to normally sized bi- or uninucleate cells. We then focused on a striking example of apparent multinucleation—a central cell with 14 apparent nuclei (labeled as Cell 1) surrounded by multiple cells lacking nuclei (Cell 2, 3, 4)—for closer inspection and 3D reconstruction (white box, Figure 1A). At traditional en face imaging angles, ZO-1 labeling alone suggested clear multinucleation based on an apical ring encompassing nuclei (Figure 1A). Apical α-catenin labeling also suggested the same arrangement, however with a somewhat thicker ring shape encompassing all but two nuclei (nuclei 1A and 1B) in Cell 1 (Figure 1B). Maintaining this viewing angle, we then generated a 3D reconstruction of all channels and zoomed in to more easily assess the objects’ spatial relationship (Figure 1D–F). With the addition of 3D information in the reconstructions, it becomes apparent that lateral α-catenin was acting as a separator between a central pair of nuclei (1A and 1B) and nuclei of the surrounding cells (2A, 2B; 3A, 3B; 4A, 4B). Given that the corresponding surrounding cells seemingly lacked nuclei under normal en face viewing, we argue that adjacent cell nuclei are instead pushing into the lateral wall(s) of the central cell, only visible when a lateral membrane marker like α-catenin is used. Viewing the reconstructions from the basal perspective (from “underneath”) further reinforced this point, showing alpha catenin forming a central ring that extends centrally down from the apical surface of Cell 1 in a pore-like or funnel-like fashion, implying the lateral walls of the surrounding cells are not orthogonally oriented to the apical surface (Figure 1H).

Video S1 (Supplemental Material Video S1) presents an isolated z-stack imaged at 100× magnification with labeling of ZO-1 (green), α-catenin (red), and nuclei (blue) from the same FOV as Figure 1. Initially oriented basal-side up, with the apical surface away from the viewer, a large, seemingly multinucleate cell is found in the top-middle of the image. The cell is much larger than normal by about 3-fold on each of the X- and Y-axes, suggesting an 8–10-fold increase in cell size. This cell has a number of nuclei around its perimeter that appear to be within the cell depending on viewing angle. Other cells of interest include those at the bottom middle, where again it appears that larger cells are multinucleated with a number of nuclei appearing near their perimeters. As playback proceeds, orientation is flipped with the apical surface being at the top when viewed orthogonally and closest to the viewer later when viewed en face. The viewer is then “flown” through the image where the auto fluorescent aggregate and laterally oriented α-catenin is visible below the apically located ZO-1 rim. Zooming closer and viewing from the apical surface, reveals multiple nuclei clustered around the perimeter of the enlarged cell. The α-catenin signal is removed to highlight the ZO-1 outline, mimicking typical RPE cell border delineation imaging. When the α-catenin channel is restored, it is apparent that the large cell is overhanging these nuclei on the ring and edge. When rotated to a side or underneath view, it becomes clear that these nuclei actually belong not to the central cell, but to the adjacent neighboring cells, and the adjacent cells have been pushed outward. We do not know yet whether it is the central large cell pushing over the top of the adjacent neighbors or if the neighbors are pulling on the central cell resulting in over-coverage of the neighboring cells.

Video S2 presents a 3D view of a region which contains greatly dysmorphic RPE cells following subretinal injection (SRI)-induced retinal detachment and recovery. Here, relying on ZO-1 (green) solely for individual cell demarcation (as is typically found in the field) is problematic as abnormally large cells are adjacent to select cells of strikingly smaller area (Figure 2). This “purse-string” pattern has been observed in multiple models of RPE stress when viewed in en face 2D images and potentially reflects cells in the process of programmed cell death and/or removal from the sheet. However, it is unclear if the larger cells are actively pushing out the smaller cells or merely expanding to fill in the previously occupied space to maintain the RPE’s barrier integrity. When imaged with concurrent α-catenin staining (red), the viewer can observe many different underlying cellular shapes that do not directly align with the ZO-1 perimeter staining. For instance, in the top-middle of the video, there is a large, spherical structure of labeled α-catenin that spreads underneath multiple ZO-1 delineated perimeters. This structure encompasses two nuclei (blue), separating them from the pair of nuclei directly below. Without this additional ultrastructural information, the viewer might erroneously conclude that all four nuclei are contained in an enlarged, multinucleate cell, when in reality it is two adjacent cells. When looking at the enlarged cell in the right-middle region of the video (just below the ZO-1 labeled cell of very small area), the viewer appears to see four nuclei encompassed by the ZO-1 perimeter. However, when viewed as a 3D aspect, it is apparent that the left pair of nuclei are visible through an open cylinder of α-catenin, suggesting that they are located in a different cell, with the pair on the right more superficially separated. Special care should be taken to determine in 3D space if these two pairs of nuclei are contained by the enlarged cell or are indeed in separate cells. Thus, when viewed in 3D with the addition of lateral labeling, this video exemplifies the multitude of dynamic and dysmorphic reorganization that occurs following RPE stress.

In addition to en face flatmount imaging, another widely used approach for assessing structural changes in RPE damage models involves imaging ocular cross-sections with cell border and nuclear staining. Although ZO-1 is commonly used to label the apical surface of RPE cells, it does not reliably distinguish the lateral boundaries between individual cells. In Figure 3, we demonstrate that α-catenin effectively labels both the basal and lateral surfaces of RPE cells—an underutilized feature that addresses a need in the field. Here, we have selected an FOV from a healthy, whole-circumferential paraffin-embedded section (Figure 3A,B) and isolated three adjacent RPE cells (white box Figure 3B) for higher magnification imaging and 3D reconstruction visualization (Figure 3C–H). Distinct morphologies in RPE nuclei (~5 µm, round/potato shapes, 5–6 speckles) versus choriocapillaris nuclei (axially thin, irregular, less speckling) are visible from a lateral view (Figure 3C,D). α-catenin labels lateral and basal faces with uniform staining and thickness, with lateral walls in adjacent cells consistently oriented at generally perpendicular angles to the basal membrane (greater than 45°). In Video S3 we show rotation of the 3D reconstruction shown in the last column of Figure 3, for alternate-angle viewing of lateral and basal face labeling. Initially starting with a single FOV highlighted in Figure 3B we then zoom into the selected ROI from Figure 3C–H and rotate through 3D space. The view is then widened to encompass the entire lateral portion of RPE/choroid interface, highlighting differences in nuclear structure and position between RPE and the underlying choriocapillaris.

In Figure 4 and Figure 5, we present images from untreated and NaIO_3_ stressed mouse RPE flatmounts. Green fluorescence indicates RPE cells expressing ZsGreen in generally high levels, partly masked in untreated RPE by the heavy melanin of the C57Bl/6J strain. The mice used in these experiments were genetically tagged using a floxed mouse line crossed to an RPE specific Cre driver line. The resulting cross yielded mice with a permanent genetic marker of RPE cells. Any RPE cell that expressed Cre from the Best1 Cre driver line was tagged as green because of the Cre driven deletion of the Stop codon from the CAG-lox-stop-lox-ZsGreen flox construct. While not all RPE cells expressed sufficient Cre, about 75–90 percent of the RPE cells did, rendering those cells as green, and that tag was permanent because of the floxed gene in the endogenous genome of the given cell. Even if that RPE cell divided, the resulting daughter cells would still be marked as green, and likewise, if the green RPE cell transitioned to any other phenotype or cell type, that cell would still remain tagged by heavy expression from the permanently encoded endogenous CAG-ZsGreen transgene in that cell’s genome. While the CAG promoter is active in virtually all cell types, it is especially active in motile, mesenchymal cell types. Other retinal cell types express different CAG promoter activity in different cell types [35], but the very bright ZsGreen protein still makes almost all cell types patently green by fluorescence microscopy of any sort.

In Figure 4, we illustrate a series of RPE flatmounts from untreated (Figure 4A,D) and NaIO_3_ treated mice (Figure 4B–F). The ZsGreen in 4A,B was partly masked by heavy melanin pigment normally found in C57BL/6J mice, but the RPE cells do display ZsGreen fluorescence. After NaIO_3_ oxidative stress, many RPE cells were killed in the central posterior third to half of the flatmounts (Figure 4B–F). Boundaries between “normal” and damaged cells have been marked on each flatmount image with colored circumferential lines.

Only some residual cell debris retained any ZsGreen fluorescence. In a mid-periphery bullseye ring, there was heavy ZsGreen fluorescence coincident with highly elongated and irregularly shaped cells that appeared to pile on top of each other. Farther more peripherally there was a bullseye zone of elongated cells with distinct ZO-1 outlines suggesting that these cells retained the characteristic RPE apical–basal polarity, cell-to-cell contact, and adherence in a single monolayer to underlying Bruch’s membrane even though elongated radially. Even farther out into the periphery to the ciliary body, the RPE cells appeared to exhibit normal shape and size, characteristic of that far peripheral zone [15].

In Figure 5, we show a close-up of RPE cells from a sodium iodate-treated mouse. The highly elongated morphological phenotype is readily apparent in the region towards the top of the image (Figure 5A). Cells with stronger green fluorescence tended to be more elongated. As elongation increased, ZO-1 staining diminished, and cells progressively lost straight edges, symmetry, and regularity. In highly elongated cells, nuclear positioning became more difficult to discern.

To evaluate morphological differences between EMT-like cells induced by sodium iodate and nearby normal RPE cells, we analyzed 30 of each from a single region of interest (Figure 5A). Cells within each group were selected based on clear border visibility and similar distances from the central damage zone with EMT-like cell selection limited by loss of border labeling in severely abnormal cells (thus introducing a potential selection bias). Quantitative analysis revealed significant differences in area, circularity, and aspect ratio between the two groups (Figure 5B).

Cell area was significantly larger in EMT-like cells (931.8 ± 68.8 µm^2^) compared to normal cells (441.2 ± 68.8 µm^2^; *p* < 0.0001, unpaired *t*-test). Circularity was significantly reduced in EMT-like cells (0.5903 ± 0.0277) versus normal cells (0.8206 ± 0.0277; *p* < 0.0001). Aspect ratio was significantly higher in EMT-like cells (2.912 ± 0.203) than in normal cells (1.749 ± 0.203; *p* < 0.0001), indicating increased elongation. Morphological characteristics of normal cells were consistent with previously published findings [45,46,50].

These results demonstrate that EMT-like RPE cells undergo marked morphological changes, including increased size, elongation, and loss of roundness—key features of epithelial–mesenchymal transition which can be used for cell type classification in future experiments.

## 4. Conclusions

The images and work presented here emphasize the need to determine the location of nuclei accurately, precisely, and reliably in RPE cells. By using relatively standard confocal microscopy but with the appropriate approach to instrumentation and image acquisition settings, we simply employ a careful examination of nuclear localization in 3D. We used Imaris, a user-friendly, commercially available program for visualization and creation of videos, however other software (including ImageJ with appropriate plugins) would likely be adequate for the same purpose.

Our work complements recent RPE studies using advanced imaging techniques like serial block-face scanning electron microscopy (SBF SEM) [25,51,52] and Structured Illumination Microscopy (SIM)/super-resolution microscopy [53,54,55,56,57,58,59,60,61,62]. While these valuable studies enhance understanding of individual RPE cell function, they typically analyze a limited fraction of cells from an entire eye. In contrast, our study aimed to utilize widely available confocal microscopy for a comprehensive analysis of nearly all RPE cells, their intercellular contacts, and their variations across the entire eye. We speculate that the details of the intertwining of adjacent RPE cells and Bruch’s membrane would be better understood at the super-resolution level. We hope that this article will stimulate the field to look more closely at nucleus positioning within RPE cell at higher resolution. The existing literature has already demonstrated RPE abnormalities in both IRD models and AMD tissue, to frame our observations in light of known pathological changes. For example, collateral RPE damage in the rd10 mouse model [27] and in Stargardt’s Disease [63,64,65] illustrates how RPE morphology and function are compromised in degenerative conditions. These examples help anchor our findings from acute disease experimental models to observed RPE disruption in human diseases, including AMD and inherited retinal diseases.

A key question when analyzing RPE dysmorphia is if neighboring cell nuclei remain in their respective origin cells but are visualized within an enlarged central cell’s borders when viewed en face, or are indeed components of a larger multinucleate cell. Our results suggest that when looking at large, multinuclear cells in an RPE sheet which has been subjected to stress, it is important to visualize these changes from a 3D perspective at the individual cell level. In multiple images, we show that nuclei which appear as members of a large multinucleate cell are actually still members of surrounding cells when viewed at higher magnification and alternate angles than the standard apical to basal collapsed view.

Correct interpretation of multiple nuclei in RPE cells depends on a better understanding of how the basal and lateral faces of the RPE cells move following aging and stress. A small proportion of RPE cells appear capable of greatly increasing in size following severe stress. Largely absent under normal conditions, it remains unclear if all RPE cells have this capability to vastly enlarge, or if only a small subclass of RPE cells can do this. Further experiments investigating damage-associated morphological abnormalities are required to understand the unusually large cell phenotype and find potential common traits, if any, between damage conditions. It is likewise unclear if these cells can undergo further morphological or functional transformations such as, but not limited to, epithelial–mesenchymal transition (EMT).

Finally, a fate-mapping strategy employing RPE cells genetically marked by a Cre-lox system allows accurate tracing of the lineages of cells of previously unknown origins in the subretinal space. We were able to indelibly mark cells of extremely elongated and irregular shapes and divine that they were of RPE origins with this strategy in mice treated by tail-vein injection of small amounts of NaIO_3_.

Limitations: Each of the three acute damage models used in this study has limitations. Most notably, (1) mice are not humans and lack a macula, and (2) these models represent acute RPE injury, whereas AMD is a chronic, late-onset disease. However, photoreceptor cells in mice are more densely packed than in the human macula [66], suggesting that the central mouse retina may better approximate the macula than previously assumed. Moreover, many RPE diseases share common etiologies, in that the RPE cells are robust in restoring many healthy functions up to a critical point of no return. Occasional, infrequent cell death and clearance may also be a general feature of epithelial sheets, mediated by similar purse-string extrusion mechanisms [67,68].

That said, as expected, all models showed rapid and obvious RPE damage within hours of insult. We have detected both irreversible obliteration of the RPE sheet in all these models, as well as some evidence of restoration and recovery of RPE cells by EMT and re-differentiation. In each model, we detected barrier recovery and partial recovery of the RPE sheet morphology, but that took months. We take into consideration that “RPE cell packing varies with their “geographic location” [15,66]. Additionally, the susceptibility of RPE cells to damage from metabolic stresses may differ depending on whether they are in a central or far peripheral position [66].

## Data Availability

The original contributions presented in this study are included in the article/Supplementary Materials. Further inquiries can be directed to the corresponding author.

## Supplementary Materials

The following supporting information can be downloaded at: https://www.biorxiv.org/content/10.1101/2024.12.04.626881v3 (accessed on 22 July 2025); Video S1: 3D video of LIRD-induced RPE dysmorphia (corresponds to Vid 1 in link); Video S2: 3D video of subretinal injection (SRI)-induced RPE dysmorphia (Corresponds to Vid 2 in link); Video S3: 3D video of RPE histological section (Corresponds to Vid S3 in link). Figure S1: Full Resolution RPE FM from OS eye following PBS injection (corresponds to Figure 4A in link); Figure S2: Full Resolution RPE FM from OD eye following PBS injection (corresponds to Figure 4D in link); Figure S3: Full Resolution RPE FM from OS eye following NaIO_3_ injection (corresponds to Figure 4B in link); Figure S4: Full Resolution RPE FM from OD eye following NaIO_3_ injection (corresponds to Figure 4E in link); Figure S5: Full Resolution RPE FM from OS eye following NaIO_3_ injection (corresponds to Figure 4C in link); Figure S6: Full Resolution RPE FM from OS eye following NaIO_3_ injection (corresponds to Figure 4F in link).
